# Interdisciplinary technology assessment of service robots: the psychological/work science perspective

**DOI:** 10.1007/s10202-012-0113-6

**Published:** 2012-11-27

**Authors:** Martin Fischer

**Affiliations:** Institute of Vocational and General Education (IBP), Karlsruhe Institute of Technology (KIT), Karlsruhe, Germany

## Abstract

The article sheds light on psychological and work science aspects of the design and utilization of service robots. An initial presentation of the characteristics of man–robot interaction is followed by a discussion of the principles of the division of functions between human beings and robots in service area work systems. The following aspects are to be considered: (1) the organisation of societal work (such as the different employment and professional profiles of service employees), (2) the work tasks to be performed by humans and robots (such as handling, monitoring or decision-making tasks), (3) the possibilities and the limitations of realizing such tasks by means of information technology (depending, for example, on the motoric capabilities, perception and cognition of the robot). Consideration of these three design perspectives gives rise to criteria of usability. Current debate focuses on the (work science) principles of man–machine communication, though in future these should be supplemented with robot-specific criteria such as "motoric capabilities" or "relationship quality." The article concludes by advocating the convergence and combination of work science criteria with ideas drawn from participative design approaches in the development and utilization of service robots.

## Abstract (de)

Der Artikel beleuchtet psychologische und arbeitswissenschaftliche Gesichtspunkte bei der Gestaltung und dem Einsatz von Service-Robotern. Zu diesem Zweck werden zunächst Merkmale der Mensch-Roboter-Interaktion dargestellt, um anschließend Prinzipien der Funktionsteilung zwischen Menschen und Robotern in Arbeitssystemen des Servicebereichs zu diskutieren. Zu berücksichtigen sind hier: (1) die Organisation gesellschaftlicher Arbeit (z. B. unterschiedliche Berufsprofile von Servicekräften), (2) die Arbeitsaufgaben, die von Menschen und Robotern erbracht werden sollen (z. B. Handhabungs-, Überwachungs- oder Entscheidungsaufgaben), sowie (3) die Möglichkeiten und Grenzen der informationstechnischen Realisierung solcher Aufgaben (abhängig z. B. von Fähigkeiten der Bewegung, Wahrnehmung und Kognition des Roboters). Bei Berücksichtigung dieser drei Gestaltungsperspektiven gelangt man zu Kriterien der Gebrauchstauglichkeit (Usability). In der Diskussion sind hier (arbeitswissenschaftliche) Grundsätze der Mensch-Maschine-Kommunikation, die jedoch künftig durch roboterspezifische Kriterien zu Themen wie „Bewegungsfähigkeit“oder „Beziehungsqualität“zu ergänzen wären. Der Artikel endet mit dem Plädoyer, bei der Entwicklung und Anwendung von Servicerobotern arbeitswissenschaftliche Kriterien mit Ansätzen partizipativer Technikgestaltung zu kombinieren.

## Introduction

There is no such thing as *the* psychological perspective in the sense of a single coherent psychological view of service robotics: in the course of the further differentiation of the science of psychology and the development and differentiation of robotics, different psychological perspectives are discernible whose representatives are in some cases not even engaged in dialogue: the psychology of work and work science investigates the task appropriateness and usability of robotics, in addition to questions of work safety; cognitive psychology models the cognitive performance of human beings and robots, psycholinguistics analyses the linguistic behaviour of human beings and technical systems; social psychology addresses the significance of robotics in society, and now, even clinical psychology is involved, where robots are deployed for therapeutic purposes, as in the treatment for individuals suffering from dementia. Last but not least, political psychology or social psychology concerns itself with the psychosocial impact on individuals replaced by robots.

In the following, the above psychological perspectives will be subsumed under a work science viewpoint, since the aim—as suggested by the term "service robotics"—is to shed light on the potential consequences of the design and utilization of robots within the context of (professional or private) service work. Undeniably, the term "service" is used in a somewhat poorly defined manner in connection with robotics and presents a number of definition problems. From a work science viewpoint, service work differs from production work in that the intended utilization value of the work lies in the service activity itself and is not necessarily manifested in a material product: the utility value of the services provided by banks lies in the consultation of customers, even if today this consultation takes the form of a document (consultation documentation) and both the bank and the customer are using the consultation to pursue further (economic) objectives. Likewise, the utility value of care activities lies in care, even if material objects are used or even new ones created (prostheses, for example). This work science viewpoint is not commensurately reflected in the area of service robotics, as the term "service robotics" is usually applied as a mere negative differentiation from "industrial robotics" (cf. Decker et al. 2011). A common differentiation is provided by the "United Nations and The International Federation of Robotics" (2002), where "professional service robots" and "personal service robots" are compared to industrial robots. In the following, we will be dealing with these two former types of robots.

## Human–robot interaction

The psychological and work science perspectives outlined above (in a general sense) are both concerned with examining and designing human–robot interaction (human–robot interaction = HRI). HRI is an area of human–machine interaction, or human–computer interaction (human–computer interaction = HCI), both of which represent interdisciplinary fields of research and development. In comparison with traditional human–machine interaction and/or human–computer interaction, the interaction between humans and robots differs—partially in principle and partially by degree—in the following aspects (cf. Habel 2009):

1. Robots act (partially autonomously) in the physical world. HRI therefore covers the joint action of humans and robots, as well as the differences in such action, and also the physical interaction of humans and robots.
2. Robots are equipped with information processing and information output systems with which robots "perceive" the physical world and "speak" about it. Consequently, HRI deals with the communication between humans and robots relating to jointly perceived objects.
3. By means of their physical presence, appearance and ways of acting and communicating, robots can give rise to a "relationship" of various types with humans. This may result in humans ascribing attributes similar to living beings to robots and may even entail entering into a relationship similar to one with a living being.
4. With the help of their "artificial intelligence^1^", their actuators and their communication capabilities, robots can execute motions, perform work tasks and establish relations that were previously attended to by human beings. Consequently, HRI in the service robotics area also addresses the relationship between robots and humans whose (service) tasks have been partially or entirely replaced by robots and who may possibly still be active within a shared work system with the robot.

As a result of the aspects cited above, human–robot interaction in the area of service robotics deals with both the potential aspects that are common to the interaction between humans (as in the relationship between service employees and clients, for example), as well as addressing actual or anticipated differences vis-à-vis interactions between human beings.

The following analysis is based on a work science perspective that covers both the area of gainful employment and the area of private service work. A task, in this context a service, is carried out to meet a certain purpose. What part of the task is assigned to human beings (either a human service worker or the client of a service) and which part to the robot? Which work processes and which technical processes contribute to achieving the respective purpose or objective? How do the processes and how does the "interface" of the robot have to be designed, and what criteria can be cited here to identify a "successful" service^2^? The analysis will focus on these questions.

## Human–robot division of functions

The design of the "interface" between human beings and robots is a central element in service robotics, considering that robots never act completely alone. They are programmed by human beings, switched on and switched off, and they are provided with information or instructions by a human being (possibly a different person). A human being (again, possibly a different individual) will listen to the robot or receive a glass of water from the robot. There is a human–robot division of functions inherent to each human–robot system which answers the question as to which tasks the robot performs and which tasks human beings handle—which is one most important design questions from a psychological viewpoint. At this juncture, the course is set in a direction which is subsequently almost impossible to reverse. This is exemplified by an example from the early days of robotics (cf. Fischer and Lehrl 1991): if a robot is to insert screws within the context of a work system and the robot is not capable of tightening them, then precisely this residual task will be assigned to human beings—one that can hardly be surpassed in terms of monotony. Albeit under different conditions—as gainful employment is not involved in many application scenarios here—the same question arises in the area of service robotics: which tasks can and should (still) be performed by human beings and which tasks should robots assume?

There are three design perspectives addressing these questions which should be considered in the development of service robotics.

Firstly, there is the *task*-*oriented design perspective.* Here, the key question is which tasks are to be processed with the help of the technical system to be developed, and which technical functionalities are to be created in this context.

The question of task orientation is embedded in the (desirable) *organisation of societal work.* This design perspective deals with the aspects of work organisation, authority to instruct and power of control, the division of labour and cooperation through to the design of tasks at the workplace or in private life, which must be considered and decided upon in system development. The term "organisation of societal work" indicates clearly that the practical problems confronting technology development and technology design not only stem from the concrete application field of the technical system but comprise societal preconditions, and the conditions and results of the application of technology (i.e. profitability, employee training and recruiting practices, the hierarchy of professions, societal and company status of service concepts).

In connection with the *information technology design perspective,* the question is addressed as to how the functions of the robotic system to be developed that are reflected in organisational terms are to be realized in terms of hardware and software technology. The realization of information technology is not derived from the other design perspectives in a one-sided manner: here, the fact should not be underestimated that the availability of (technical) resources will also give rise to the creation of (new) functions.

All three design perspectives are closely interlinked, but will be discussed sequentially in the following for the sake of clarity. As an example of the reciprocal contextual impact here, one may consider the fact that a service robot assisting handicapped persons in the home should be equipped with a language processing system (information technology perspective) in order to be able to respond to instructions given by the handicapped person for the intake of food (task perspective). This also applies to the application scenario—which may possibly present much higher demands—in which the robot’s repertoire of activities (conventionally via programming) is to be extendable by the care provider active in the household—and not only by way of a trained programmer to be called in specifically (organisation perspective).

### Perspective of the organisation of societal work

In most cases, the closely defined psychological analysis of human–technology interaction wrongly excludes the perspective of the organisation of societal work. It is here, however, that questions arise whose answers will have considerable consequences for all further development and application steps—for example, at the individual level, whether service work consists exclusively of the instrumental performance of a desired function (as in providing a handicapped individual with food, for example) or whether the service is implicitly or explicitly connected to other wishes or demands (for example that the service employee "understands" the handicapped person or that the service employee "notices" that the handicapped person is experiencing acute health problems). In the case of the first option cited (instrumental provision of the desired function of "food intake"), it might be irrelevant as to whether a robot or a human service employee fulfils the function. In the case of the second option, it is not irrelevant, but it cannot be automatically stated that only a human being can fulfil such wishes—this depends on the robot’s performance capabilities (in medical diagnostics, for example) and the reception of the client (for example, as to whether the client feels "understood" by a robot).

An additional aspect is the fact that not only the demands of customers and clients count in the service work area. Services in the care area have already been very clearly defined and delimited from the point of view of profitability to the extent that there are no objections to the utilization of robots—provided, however, that such connections of care services with economic considerations are deemed acceptable. It is for the same economic considerations that the use of robots is not regarded as an option, independent of technical problems, as long as the (low) wages in the care sector do not promise an expedient deployment of robots. In turn, these profitability considerations—at least in the field of care—are overshadowed by legal obligations associated with care services and the question as to whether sufficient labour resources are available. If this is not the case, and other forms of labour recruitment (foreign labour, retraining measures, etc.) prove unsuccessful, the use of robots in the field of care would be a limited option from this perspective—provided that all technical problems have been solved. "Limited option" means that service robots would presumably interact within a work system alongside human care providers and would interact with human beings (care staff, as well as clients/customers) who are unfamiliar with how to operate a robot.

What we have roughly outlined here for care services could also be applied to other service areas. The following aspects should always be considered (please refer to Ott for the economic aspects, and to Dreier & Spiecker genannt Döhmann in this volume, for the legal aspects):

- the expectations (requirements + demands) of customers/clients, as well as solvent demand and political feasibility;
- the capabilities, work performance and remuneration of the service employees active to date, as well as the reservoir of existing labour;
- profitability considerations (and potential obligations) of service employees, as well as
- the performance capability, procurement and maintenance costs of robot technology.

In addition, when service robots are deployed in a private environment, robot application strategies may deviate to a certain extent from the expenditure and profitability considerations mentioned, while the personal preferences and interests of robot users will enter into the picture. In order to determine a given or anticipated societal need for the deployment of service robots at the universal level, the respective relevant professional or private work or relationship system would have to be recorded and analysed according to the above aspects. As already mentioned, "service" is a comprehensive and also somewhat diffuse term under which many different services can be subsumed. Is a diving robot that retrieves the silver treasure of sunken ships from the depths of the oceans a service robot? In cases in which the performance capability of the robot exceeds human performance capabilities, as well as in cases of deployment in dangerous or health-damaging environments, the use of robots can be desirable in societal terms and also worthwhile in individual cases, and the robot will operate largely in isolation. In all other instances, robots in the service area will take on functions previously performed by human service employees. At present, and this is likely to apply to many service areas, it is only discernible to a very limited extent as to what incentives are given for the key protagonists to deploy robots. Apart from experimentation, demonstration or game purposes, service robots will initially be deployed—if at all—only sporadically and together with service employees. This means that the division of functions between humans and robots in the service area must be considered in terms of two aspects:

- The division of functions with the service employee;
- The division of functions with customers/clients

### Task-oriented design perspective

In determining which tasks in a given service area are to be performed by service employees, which by the customer/client and which by robots, the first question that presents itself is: who decides this? This question arises due to the fact that today’s robots are equipped with AI-based systems enabling them (within a limited framework, in other words semi-autonomously) to make decisions. Here, the issue at hand is the interaction between human and artificial intelligence: when is a robot permitted and intended to autonomously perform a service based on the diagnosis of a situation without having received an explicit command? When is a robot allowed to correct faulty human action, even without explicit instructions? This is a psychological issue, as questions of judgement capabilities and mental performance play a role here, while it also extends into the ethical and legal dimensions of technology assessment.

If one initially only considers the psychological aspect of human judgement versus artificial judgement, the following question arises: how does the functional mode of an AI system differ from human decision-making (cf. Fischer et al. 1995, 1996; Fischer 2000, p. 158 f.) ? Human beings make decisions on the basis of knowledge (such as rules derived from an area of expertise), experience (recollections of similar decision-making situations in the form of images or language), emotions (such as feelings as to whether a solution is good or not) and motivation (linked to the expectation of being able to arrive at a solution in the first place). AI systems lack emotion and motivation, while knowledge and experience are modelled in a certain manner. The description of functional contexts within the problem area to be assessed serves as knowledge. In terms of experience, case examples, statistics, etc., can be subsumed. Moreover, attempts are made to reconstruct experience-guided action by assigning different probabilities to conclusions that the system AI is to make under the conditions entered in advance.

Taken as an individual aspect within the context of the AI-based judgments, both the description of functional contexts and the input of case descriptions far surpass the reception and recollection capacities of human beings. The computer-based combinatory handling of function and case descriptions exceeds the capabilities of the human brain in terms of processing speed. As everywhere in the utilization of computers, high storage capacity and data processing speed are also key features of AI systems. An AI system, however, cannot establish a content connection between knowledge/experience and the current problem situation, only a formal context: the inference mechanism of the AI system diagnoses concordance or non-concordance of a problem based on a canon of rules, case descriptions, statistics, etc., conclusions are drawn and the respective robot actions are generated.

This may not pose a problem in the case of a manageable number of action situations and alternatives. The number and variety of dishes to be dispensed at a McDonalds outlet would essentially be manageable. Certain customer responses, however, would be less predictable: what does a service robot do if a customer is angered because of the quality of the food and pours beer over the robot?^3^ A short circuit would not be a particularly appropriate response here.

To return to the core of the problem: in many service environments, the action situations and alternatives are less standardised than in McDonalds outlets.^4^ In the case of the presence of customers/clients in the service area, the number of potential problem situations is essentially unlimited and can merely statistically (as a probability) but not actually be anticipated. Ideally, human service employees master such imponderables by their capability to recognize the general aspect of a situation ("Mr X has a cold") as well as the specific aspect of a problematic situation ("But this cough is cause for concern"). The *simultaneous grasping and retaining of identity and difference* of an issue practiced quite naturally in human thought is the essential distinction from machine learning as realized in AI systems. By comparison with conventional programming, the inference-controlled combination of information units and procedures in artificial intelligence programmes does achieve a considerable dynamic. The information unit itself (such as a judgement) is statistical in nature, however, which means that in a digital computer, it must ultimately either refer back to state 0 or state 1.

The capability to *simultaneously* grasp and retain the identity and the difference of a matter provides the basis for the "shapes of terms" *("Begriffsgestalten*"*)* as described by Volpert (1988, p. 180 f.) that enables creative action.

“We also recognize recurring moments in situations and act in a repetitive manner in such situations. These are not mechanical sequences, however, but shaping processes set against the background of our living environment. The foundations of what is recurrent in our perception and action are flexible underlying patterns, shapes of terms. They have a clear and concise structure, while leaving space for variations of what is concretely given in the particular instance. We know what an “apple” is or the act of “giving” but in each situation we perceive a special “apple” and we act in different ways accordingly. The structure remains clear and distinct, while the individual case varies. This enables permanence and stability without rigidity. (…) We acquire such basic patterns as a cultural heritage and develop them further based on our own experience, also applying them to other areas of life. This is the essential foundation of human creativity.”

The line of argumentation presented whereby the simultaneous grasping and retaining of the identity and disparity of an object being the crucial difference between human and the artificial intelligence of a digital computer is largely also supported by the "analysis of AI-machines from the viewpoint of theories of meaning and theories of time (D’Avis 1994). According to the central argument that D’Avis (l.c. p. 351) presents," "the essence of mind lies in the assigning and recording of meaning. Meaning itself can only arise in a three-way relation between the world, language and substrate under the (…) preconditions of temporal isomorphism." Accordingly, thought constitutes meaning arising through the difference between characters and signage and that which is signified (language and world). This difference is experienced through the experience of time in the world and it can only be experienced given that the substrate of thought, the brain, is itself subject to temporality. To return to the starting point, the simultaneous grasping and retaining of the identity and non-identity of an object calls for an awareness of time; in the present perception of an object, the subject knows its past and anticipates its future, while the computer is always in the present.

The situation-based capability to interpret given judgements and conclusions is what characterizes human thought as compared to a merely linking of cause and effect according to formal logic. This is where the capability of living intelligence is rooted to question given judgements and conclusions and recognize their validity and scope—by comparison with the automatic processing of problems within the context of AI-based diagnostics, which does not possess the attribute of self-reflection.^5^

The differences between machine-based knowledge processing and living thought processes presented here mean that a service robot has no awareness or consciousness of whether it is in danger itself or could endanger others. A service robot working in the household and assigned to provide food for a handicapped person could ideally, with the help of its AI system, differentiate beverages from detergents and select appropriate action options. However, the robot is not capable of thinking: "I could possibly poison someone."

From a work science point of view, the lack of self-reflection of the robot means that the potential risk posed by a partially autonomously operating robot is high and that appropriate control mechanisms and safety measures have to be installed. Initially, these safety measures could consist of limiting the robot’s options for taking action in relationship to human interaction partners. In the treatment for individuals suffering from dementia, a robot is used that resembles a little seal, can emit seal-like noises, wobble its head and roll its eyes—whereby all of these responses can be geared to the reactions of the contact person suffering from dementia. But the robot cannot bite like a real seal. In the case of a service robot working in a household, however, actuators are necessary (such as robot arms, for example) which pose a potential risk. The specific issue of the speed with which robot arms move towards the client is itself the subject of many different experiments, as a robot is not capable of thinking: "I could frighten someone." As outlined, a robot also has no awareness or consciousness as to whether motions that do not frighten one person might alarm another individual.

With regard to the human–robot division of functions, all this means that human beings (whether service employees or clients) must have the option of stopping the robot’s actions at any time and overruling the robot if necessary, since in the few standardised work environments that many service areas offer, sufficiently safe robotic action cannot be ensured ex ante.

The considerations presented address the question: who makes the decisions? They do not yet contribute to answering the question as to which qualities technical support systems—such as service robots—possess and should possess as such and in a complementary function to human capabilities. By way of a contrasting analysis of tasks (cf. Volpert 1987; Dunckel 1997), a useful technical system does not merely represent the most true-to-nature reflection of what users know themselves, do and are capable of doing. Far more, it is a matter of a quality of information technology that complements user competencies.

The strength of robotics lies in the precise execution of operations that allow a certain measure of standardisation, even under conditions unfavourable to human beings in terms of time and place, while also presenting a certain breadth of variation. If the tasks were largely unvaried, the question arises as to what the robot’s flexibility would be required for: presumably a dishwashing machine is more suitable for washing dishes than a robot. This is one side of the coin. The other side of the coin is the fact that if the processing of tasks cannot be standardised at all, the robot soon meets its limitations. Attending to small children, at least where more than mere monitoring is called for and an understanding response to children’s behaviour is required, exceeds the current and foreseeable performance capabilities of service robots. Within this bandwidth, a range of tasks is conceivable for service robots that are either strenuous for human beings to perform (entailing negative impacts on health), are not in line with human time schedules (night work) or exceed the performance capabilities of elderly, sick or handicapped persons.

This applies to the division of tasks with clients as well as to the division of tasks with those service employees remaining in the work system. With regard to the retention of competencies and the development of service employees’ competencies, it must be ensured that the respective cognitive performance is not substituted by robots. From a work science viewpoint, the principle of complementarity in the division of tasks between humans and robots applies here too, meaning that robots take on tasks in those areas where humans are either not capable of performing the tasks, or else only with considerable difficulties.

### Information technology design perspective

Within the context of interdisciplinary HRI, the information technology design perspective is usually part of the work performed by engineers and IT specialists and will only be addressed briefly here. The key question is firstly how these considerations of the human–robot division of functions are to be realized by the respective hardware and software components, and secondly what potential arises in the division of tasks based on technological advancements.

In view of the considerations outlined above, emergency off and overrule functions must be realized in terms of information technology as a matter of principle, given the risk potential of partially autonomously operating service robots.^6^ Special possibilities with regard to the division of tasks derive from the optical, acoustic and haptic signal processing systems that robots are increasingly equipped with. This could enable users to program robots by way of natural speech or special teaching methods (such as demonstration-imitation). Some progress has been made in the programming of robots in recent years, enabling the robots to be controlled by manual operating devices ("gaming console") (Klempert 2012), the manual guidance of robots (Mentgen 2011), the utilization of tablet computers (Innovationsagentur für IT und Medien 2011), as well as imitation learning (Institut für Anthropomatik 2012) and speech commands (Hochschule Ulm 2012). All this facilitates the flexible deployment of service robots also by technically unskilled persons. At the same time, elements have been developed such as safety light curtains, trunk-like robot arms, foam protective layers for robots with food sensors with which robot movements can be stopped depending on human action or slowed down. These measures serve to improve safety in work systems shared by humans and robots below the emergency-off level (cf. Mentgen 2011, for example).

## Usability

According to the answers to the basic human–robot division of functions, as well as the answer to the specific division of tasks, (ergonomic) topics must be addressed from a psychological point of view that is part of human–machine communication. Problems of human–machine communication are not only motivated by a desire for standardisation. Entire research areas are confronted with the problem of referencing the technology understanding (Fischer 1995) and the operating modes of users of technical systems (cf. Bullinger 1985, for example; Brauer and Wahlster 1987; Nake et al. 1988). This problem is so urgent because naturally the computer-based processing of material and information flows in production and service areas does not adequately reflect the work process (Coy 1987). Within the context of software development, therefore, a translation of internal computer information processing is required that makes the programme sequence transparent and controllable in the first place. Consequently, engineers and IT specialists are not only confronted with the question as to how technical programme-related issues are to formulated in terms of language, but also and indeed primarily, which conceptual world and which action options of the users of technical systems can they reference? This also applies particularly to service robotics.

In this context, insights and findings from the area of software ergonomics^7^ are available. "Software ergonomics is the adjustment of work conditions in human–computer interaction to the sensorial-motoric and cognitive capabilities and processes of human beings." (Wandmacher 1993, p. 1) With regard to interactive media, an ergonomic structure is always assumed, in other words the design^8^ of the interactive computer system in line with human- and tasks-related requirements—in short, usability. Usability is to reflect the extended view of tools and media and is regarded as the product of effectiveness, efficiency and satisfaction (cf. Hartwig 2007, p. 21 ff.). This means that usability is not an exclusive feature of a robot, but derives from the interaction between humans, tasks and robots, influenced by a multitude of contextual factors.

In the planning of human–computer communication processes and their subsequent evaluation, Hartwig (2007) cites the principles of dialogue design according to ISO 9241 Section 10 (1996) from which the following dialogue principles are derived as the quality demands made of an interactive dialogue between humans and machines: task suitability, self-descriptiveness, controllability, conformity to expectations, fault tolerance, the possibility of individualization, and supporting of learning (cf. ibid., p. 57 f.).

- Task suitability: "A dialogue is suitable for a task if it supports users in accomplishing their work task effectively and efficiently."
- Self-descriptiveness: "A dialogue is self-descriptive if each individual dialogue step is immediately comprehensible through feedback from the dialogue system or an explanation is given on user request."
- Controllability: "A dialogue is controllable if the user is able to launch the dialogue sequence, as well as influence its direction and speed until the objective has been attained."
- Conformity to expectations: "A dialogue conforms to expectations if it is consistent and concurs with the characteristics of the user, for example the user’s knowledge of the area of work, his or her training and experience as well as generally acknowledged conventions."
- Fault tolerance: "A dialogue is fault-tolerant if in spite of discernible faulty user input the intended work result can be achieved either with or without a minimum of correction input on the part of the user."
- Individualisation: "A dialogue can be individualised if the dialogue system permits adjustments to the requirements of the work task, as well as to the individual capabilities, skills and preferences of the user."
- Supporting learning: "A dialogue supports learning if it helps the user to learn the system or guides him or her through it."

With regard to the cited criteria, the focus is clearly on dialogue with computer program via the screen. In terms of service robotics, these criteria would have to be augmented by aspects and characteristics, which also comprise the robot’s hardware and software technology components, as well as the robot’s "relationship quality." At least to some extent, the criteria for hardware and software technology components of a work system shared by robots and humans can be drawn from the ISO 10218 industrial robot standard, which contains information on design and construction demands and protective measures for operating elements, as well as the demands made on safety-related control systems, robotic halt functions, operation at reduced speeds, operating modes, demands made of collaborative operations, protection in the case of singularity, axis limitation, motion without drive, arrangements and safeguards for lifting and electrical plug connections.^9^

The following criteria were proposed for assessing and designing the "relationship quality" between humans and robots (Heerink et al. 2009):

- Trust: The belief that the system performs with personal integrity and reliability.
- Perceived Sociability: The perceived ability of the system to perform sociable behaviour.
- Social Presence: The experience of sensing a social entity when interacting with the system.
- Perceived Enjoyment: Feelings of joy or pleasure associated by the user with the use of the system.
- Intention to Use: The outspoken intention to use the system over a longer period in time.

Nowadays, the criterion of "intuitive" handling and use is a significant topic in attempting to make a technical system usable, as in the context of mobile telephony, for example. The above-mentioned methods of controlling robots also use this term. In the service robotics area, this issue assumes a particular significance: the argument of "intuitive" handling and use addresses the "appearance" of the robot, and humanoid robot systems enter the picture. People tend to personalize objects and therefore also technology. This also presents the question as to how humanoid a robot system should be for a certain task, which, as is the case here, represents a service task and is also performed inside the private sphere of humans. Attempts are made to create relationship quality, encouraging human empathy for the robot, by enabling robots to recognize facial expressions and imitate them (Gonsior et al. 2011). However, findings in connection with the "uncanny valley" (MacDorman and Ishiguro 2006) suggest that this is also possible to go "too far" in terms of human-like design, with the cooperative appearance reverting to an "uncanny" image for the human observer, which does not encourage cooperation in a major way and proves counterproductive for usability.

## Methods of technology development and technology design

How can the usability of service robots be ensured? The appropriate methods of technology development and technology design have long been discussed in work science.^10^

The intention to develop a user-friendly robot for the service area undeniably reflects a twofold contradiction (cf. also Corbett et al. 1991).

On the one hand, the assessment of a technical system as suitable or unsuitable implies a given technology. The question as to the suitability and expediency of technical support cannot be adequately decided at the beginning of the development process—on the drawing board, so to speak. At the same time, this quality should be incorporated in the result of the technology development. This contradiction points to the inadequacy of a *linear* development process to achieve the targeted objectives.

Secondly, it is not the developers themselves who are solely in the position of qualifying a technology as adequate or inadequate. If it is to be taken seriously, the claim to develop a work-oriented or usability-based technology entails evaluating a technology based on the well-founded vote of the users. Those who are qualified for technology development do not possess—or not exclusively at least—the competence to measure their developments by the benchmark of usability. And those who are attributed the competence for technology assessment and evaluation are generally not qualified for development activities. This contradiction shows up the inadequacy of a *one*-*dimensional* development process exclusively controlled by engineers or IT specialists.

The 1990 s in particular saw a flood of publications presenting positions on the principles and development paths in the fields of technology development and technology design (cf. for example Coy et al. 1993; Rödiger 1993; Brödner et al. 1991; Ackermann and Ulich 1991). All in all, the positions taken can be assigned to the two opposing poles of the outlined contradiction: the one pole consists—in its ideal–typical expression—of postulating the scientifically determined criteria as well as the engineering standards that should be considered in technology development. The consideration of well-founded work science findings, principles of software ergonomics, so-called style guides, provide a guideline here.

The other pole is characterized by the demand for user participation—actual or potential users of technical systems should be involved in the development process at the earliest possible point in time. The position presented in this paper advocates a convergence of these two demands, which at first sight would appear to be mutually exclusive.

It is definitely not the case that the emphasis on participative approaches in technology development and technology design would render scientific research and professional development more or less obsolete—as if technology developers would only have to be told by future users what the technical system to be developed should look like. It is the task of scientific analysis and design to initially independently examine the problems and deficits in the organisation and performance of work activities. Subsequently, the investigation results and improvement suggestions are presented to the relevant protagonists in a form that enables them to review their work experience to date in an adequate manner and build on these findings in developing solutions (cf. Fischer 2001). One key factor (among others) in such a development process consists of developing and presenting principles (such as the above-mentioned principles of dialogue design) which, from a scientific perspective, are to be considered in technology development.

As these principles are open to interpretation, and interpretations with regard to the technical support of work tasks can only be provided by the technology users themselves, the participation of service employees, clients and customers of service robotics is also significant. Compagna und Derpmann (2009) refer to an approach that is not solely geared to acceptance, that is, the affirmation of technology development already intended: it is referred to as "functional-participative technology design", an approach which has been tested in the service robotics field.

## Summary

Tremendous growth rates have been identified for service robotics (cf. Thrun 2004, p. 7). This applies mainly to experimental settings, however, and less to real-life applications. In the case of long-term, permanent applications in the real world, the following open questions arise from the work science perspective:

- In the area of production work, the product constitutes the work performed. It is the manifestation of past, solidified work that is visible here. Given a certain quality level, however, a pair of trainers do not reflect whether their production involved Turkish women in Franconia, Germany, low-wage workers in China, child labour in Africa or robots somewhere else in the world. By contrast, in the service work area, work is manifested in the service activities themselves. This means that the customer/client is usually in contact with the persons or organs that perform the service work or the respective operations. This situation entails a number of implications in terms of the design and utilization of service robots, ranging from safety questions through to the "relationship quality" of services. At best, these implications have been investigated in terms of individual aspects, but have yet to be subjected to systematic empirical investigation.
- The following considerations are also relevant here: service tasks cover a wide area of private and public life, from the sorting of waste and recycling through to the supervision of infants and young children and the care of individuals suffering from dementia. Presumably, these tasks are not of equal value to most people and it is therefore not irrelevant who performs these tasks. Consequently, it is not so much a question of the societal acceptance of service robots in general, but far more that of which specific service tasks this would involve.
- An evident trend in the robot development field is towards making service robots as similar to humans as possible, that is, to imitate human looks as well as emulating mental/physical human performance. The more logical line of development from a work science perspective of developing robot functions based on contrasting task analyses that are complementary to human performance capabilities is far less evolved. Accordingly, with regard to the service area, there are no systematic investigations to date as to how robots could take over tasks in those areas where humans can only perform the relevant tasks with difficulty or not at all.
- In addressing the question of the usability of service robots, criteria can and should be applied which have been discussed in connection with the idea of "usability". These criteria, however, must be augmented by characteristics and features which firstly cover the hardware and software technology components of the robot, and secondly by those that pertain to the "relationship quality" of the robot.
- In addressing the question of user behaviour in the interaction with service robots, the majority of investigations conducted to date have confronted potential users with prototypes or images presented by robot developers. Whether future service robots would look like these prototypes and which functions they would possess if users in a specific service area were involved at the earliest possible juncture in the development process is not only an open question in terms of science. Initial approaches to answering this question have been developed with the help of a real robot prototype and potential users, drawing on selected service tasks at the "Cognition for Technical Systems" Cluster of Excellence in Munich (Scheibl et al. 2012).

## Footnotes

1 There is no uniform definition of “artificial intelligence (AI)”. With regard to the contexts discussed here, it is relevant that the robots equipped with AI systems have a knowledge base and sensor systems to record environmental data. Relationships between environmental information and the knowledge base are established with the help of an inference mechanism. In this manner, the robot is able to make “decisions” without the individual decision-making paths and the results of decisions having been anticipated by the programmer of the AI system.

2 It cannot be denied here that it is not easy to determine a "successfully performed service", as the possibly contradicting interests of service employees and clients are also faced with professional standards (cf. Dreier & Spiecker genannt Döhmann in this volume).

3 In fact, robot vandalism is being regarded as a problem to be reckoned with in the field of service robotics (Salvini et al. 2011, p. 66), and this is absolutely justified, as security issues that are difficult to anticipate may result from it.

4 Cf. Ott in this volume on problems of norms and standardisation.

5 Naturally, the capability for self-reflection does not prevent humans occasionally arriving at incorrect judgements, just as, generally speaking, the outlined differences between human and artificial intelligence do not allow any statements on performance capability with regard to certain tasks: presumably robots outperform human beings in reading barcodes—as opposed to human beings' capability of knowing their own "self".

6 Following the triggering of such functions, one of the difficulties consists in predicting the subsequent concrete operations, and balancing them between humans and robots: imagine the case of a robot functioning as a driver assistance system in a vehicle that is switched off by a user while travelling at full speed!

7 "The research and scientific analysis of interactions between humans and work is the object of ergonomics" (Schmidtke 1976, 1).

8 "‘Humanly adequate’ or ‘humane’ design presupposes an understanding of human beings and will usually only apply to a certain part of humanity. It is a necessary but not adequate criterion for the assessment of actual usability. ‘Adequacy or appropriateness for a task’ can only serve as a criterion for an aid provided (tool or medium) if the task is known, as well as the resulting activities, action and operations". (Hartwig 2007, p. 21).

9 Cf. Austrian Standards Institute/Österreichisches Normungsinstitut (ON) (2011).

10 In this context cf., the partially overlapping, partially contingent considerations from the area of technology assessment (Decker in this volume).
